# Exploring the Interplay of Genetics and Nutrition in the Rising Epidemic of Obesity and Metabolic Diseases

**DOI:** 10.3390/nu16203562

**Published:** 2024-10-21

**Authors:** Sylwia Górczyńska-Kosiorz, Matylda Kosiorz, Sylwia Dzięgielewska-Gęsiak

**Affiliations:** 1 Department of Internal Medicine, Diabetology and Nephrology, Faculty of Medical Sciences in Zabrze, Medical University of Silesia, 40-055 Katowice, Poland; 2 Students’ Scientific Association by the Department of Internal Diseases Propaedeutics and Emergency Medicine, Faculty of Public Health in Bytom, Medical University of Silesia in Katowice, 41-902 Bytom, Poland; matylda.kosiorz@gmail.com; 3 Department of Internal Diseases Propaedeutics and Emergency Medicine, Faculty of Public Health in Bytom, Medical University of Silesia in Katowice, 41-902 Bytom, Poland; sgesiak@sum.edu.pl

**Keywords:** obesity, metabolic diseases, genes, genetic risk, weight gain, BMI, dietary choices, epigenetic factors, nutrient intake

## Abstract

**Background:** Obesity has become a significant global health issue. This multifaceted condition is influenced by genetic, environmental, and lifestyle factors, significantly influenced by nutrition. **Aim**: The study’s objective is to elucidate the relationship between obesity-related genes, nutrient intake, and the development of obesity and the importance of other metabolic diseases. **Methods**: A comprehensive literature review spanning the past two decades was conducted to analyze the contributions of genetic variants—including *FTO*, *MC4R*, and *LEPR*—and their associations with dietary habits, highlighting how specific nutrients affect gene expression and obesity risk and how the coexistence of metabolic diseases such as type 2 diabetes and osteoporosis may modulate these factors. Moreover, the role of epigenetic factors, such as dietary patterns that encourage the development of obesity, was explored. **Discussion and Conclusions**: By understanding the intricate relationships among genetics, nutrients, and obesity development, this study highlights the importance of personalized dietary strategies in managing obesity. Overall, an integrated approach that considers genetic predispositions alongside environmental influences is essential for developing effective prevention and treatment methodologies, ultimately contributing to better health outcomes in diverse populations.

## 1. Introduction

**Figure 1:**
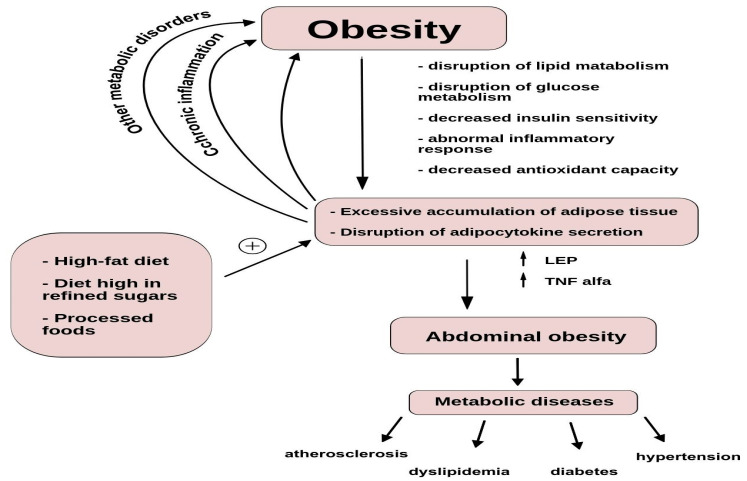
The multifaceted origin of obesity. The figure shows the importance of various factors that simultaneously affect the development of obesity and are responsible for its progression. The importance of diet, chronic inflammation, and other metabolic dysfunctions in the development of obesity, which consequently through abdominal obesity lead to other metabolic disorders, is indicated. the arrow indicates the direction of changes, and the plus signifies additivity.

As indicated by the World Health Organization (WHO), obesity is one of the most significant global health issues. The rates of obesity have nearly tripled since 1975, with over 1.9 billion adults being overweight in 2016, including more than 650 million who were obese. Globally, over 39% of adults are overweight, with 13% of them classified as obese [1]. This trend is particularly pronounced now, following almost four years of the pandemic [2,3,4]. Obesity prevalence is affected by changes in diet, lifestyle, and genetics. While the prevalence varies by region, developed countries generally have a majority of the population affected by obesity associated with excess energy consumption [5,6,7]. This suggests that nutrition plays a crucial role in obesity development. Developing countries are also experiencing a rising trend in overweight and obesity rates due to urbanization and the adoption of unhealthy lifestyles. Obesity is a persistent medical condition with a multifaceted origin, linked to disrupted lipid and glucose metabolism, diminished insulin sensitivity, abnormal inflammatory reactions, and reduced antioxidant capability [8]. The condition is characterized by an excessive accumulation of adipose tissue, which can lead to the dysregulation of adipocytokine secretion. Certain dietary patterns, such as a high-fat diet or a diet high in refined sugars and processed foods, have been shown to increase levels of pro-inflammatory adipokines like leptin and tumor necrosis factor-alpha (TNF-alpha), while decreasing levels of anti-inflammatory adipokines like adiponectin. These changes in adipokine levels can lead to chronic inflammation, insulin resistance, and other metabolic abnormalities, which are key factors in the development of obesity [9]. What is more, the excessive accumulation of visceral fat, known as abdominal obesity, poses a significant risk for various complications such as hypertension, diabetes mellitus, and dyslipidemia, and can ultimately lead to conditions like atherosclerosis, ischemic heart disease, stroke, peripheral artery disease, and certain types of cancer, notably colorectal cancer [10,11,12]. The relationships affecting obesity, its progression, and the consequences associated with it are shown in Figure 1.

There are believed to be multiple factors that contribute to the development of obesity, including environmental and genetic factors. The environment can influence genes through a process known as epigenetics, where external factors can change the way genes are expressed without altering the underlying DNA sequence. This can impact various aspects of health, including weight gain. What is more, the consumption of certain nutrients may impact the expression of many genes and may be influenced by dietary intervention to modulate obesity risk. Carbohydrates can influence the composition and function of gut microbiota, which can in turn affect obesity-related genes [13]. Certain carbohydrates, such as prebiotics, can promote the growth of beneficial bacteria that may help regulate metabolism and reduce inflammation [14]. Proteins can influence obesity-related genes in several ways: regulate energy expenditure and metabolism, control appetite, have an influence on hormones and antioxidant enzymes, up-regulate genes that promote fat metabolism, and down-regulate genes involved in fat storage [15]. Dietary fats regulate energy balance and metabolism, leading to an increase in fat storage and weight gain, and can promote inflammation and oxidative stress in the body, which can lead to changes in gene expression that promote the development of obesity [16]. On the other hand, unsaturated fats, particularly omega-3 fatty acids, have been shown to have anti-inflammatory and metabolic effects that can help regulate gene expression and prevent obesity [17].

Studies have shown that trace element deficiency can lead to an increase in adiposity and changes in gene expression related to obesity [18,19]. Trace minerals play a role in several metabolic processes, including antioxidant enzyme activity, energy production, and lipid metabolism. Key antioxidant enzymes consist of superoxide dismutase, catalase, peroxidase, glutathione transferase, and glutathione reductase and it was shown that the increased activity was associated with the up-regulation of the genes responsible for “energy expenditure” [20]. On the other hand, obesity-related genes, their expression, and/or their function each interact with non-enzymatic antioxidants: glutathione; vitamins A, C, and E; carotenoids; tocopherols; and tocotrienols [21]. Environmental factors such as diet, physical activity, and lifestyle choices play a significant role in the development of obesity but also genes themselves have an influence on body composition and weight gain. Some of the genes that have been linked to obesity include

- The *FTO* gene (variants of this gene have been associated with appetite, which is related to an increased body mass index) [22];
- The *MC4R* gene (known to play a role in regulating energy intake and appetite and metabolism) [23];
- The *LEPR* gene (encodes the leptin receptor, which plays a key role in regulating food intake, energy balance, and body weight) [24];
- The *PPARG* gene (plays a significant role in adipogenesis and metabolism regulation) [25];
- The *TAS2R* genes (regulate taste and its variants may play a role in obesity) [26].

Understanding the complex interplay between nutrient intake, weight gain, and genes is essential for developing effective strategies for preventing and treating obesity. By taking into account both genetic and environmental factors, clinicians can develop personalized interventions that address the root causes of weight gain in individual patients. Thus, the aim of the study is to describe interrelation between obesity-related genes, nutrient intake, and development of obesity.

## 2. Materials and Methods

**Figure 2:**
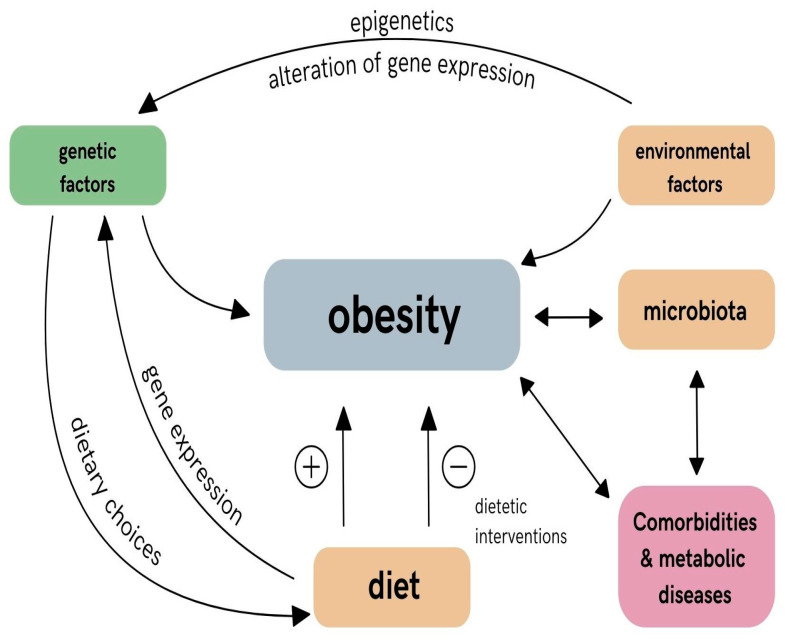
Factors modulating the obesity phenotype. The diagram shows the complex interplay between genetic factors, environmental factors, diet, and the microbiota and the development of obesity and metabolic diseases. Genetic factors influence both the predisposition to obesity and dietary choices. Environmental factors such as lifestyle and physical activity directly modulate the development of obesity. The importance of the microbiota has also been shown to increase the risk of comorbidities such as diabetes or osteoporosis. The role of diet is complex as it can act both positively and negatively by influencing gene expression. Dietary interventions affect body weight, microbiota, and metabolic health. Epigenetic mechanisms can further alter gene expression, amplifying the impact of environmental and dietary factors on the development of obesity. The arrows indicate the direction of changes, the plus indicates an increase and the minus indicates a decrease.

A comprehensive literature search was carried out using the keywords “obesity related-genes”, “nutrition”, and/or “obesity”. The selection of genes for review was based on their widely documented role in obesity research. Genes such as *FTO, MC4R, LEPR,* and *PPARG* have well-defined roles in regulating appetite, metabolism, and fat accumulation, making them central to obesity research. The second selection strategy was based on the consideration of the diverse genetic background of obesity, which is important for further therapeutic management, and therefore syndromic, monogenic, and polygenic obesity-related genes were searched for (Table 1 and Table 2). The third search strategy included genes that could influence dietary choices. This group included the *TAS2R* gene family, which, although less well known, was included because of its importance in taste regulation. The regulation of taste directly influences food preferences, which can consequently lead to the development of obesity. The fourth search strategy aimed to search for epigenetic factors that could influence the therapeutic effect and modulate the effectiveness of obesity treatment. In our search strategy, we also included genes that appeared in studies (e.g., *PLIN1*, *SIRT1-7* gene family), allowing a broader picture of the interaction between genes, diet, obesity, and their impact on comorbidities. The interrelationships between the different factors and the obesity phenotype, taking into account their modulating effect on genetic factors, are shown in Figure 2.

Available full texts and the reference lists of the relevant studies were reviewed from the past 20 years, prior to June 2024, by assessing the PubMed and Google Scholar databases. Furthermore, manual searches were conducted to identify any articles that may have been missed. This process was complemented by a search for the gray literature, and all findings were analyzed narratively. Duplicate articles were removed from consideration.

All figures in the manuscript were created by Affinity Designer v:1.10.8 license no. ARGGGP6ZR7 and Canva Pro (1.94.0) license no. 04296-35658555 for this article.

## 3. Obesity and Its Genetic Determinants

Weight regulation is influenced by both genetic and environmental factors [27]. The heritability of obesity is estimated to be between 40% and 70%. Based on studies of twins, it has been estimated that genetic factors influence BMI more strongly in children than in adults. It is therefore appropriate to recognize and evaluate the genetic basis of obesity in the physiological and molecular mechanisms involved in weight control [28]. Obesity should be considered a complex metabolic disease manifested by excessive body fat, which poses a serious health risk. It is a public health threat worldwide. It is considered a risk factor for the development of metabolic disorders, including type 2 diabetes, cardiovascular disease, and non-alcoholic steatohepatitis.

Long-term overnutrition causes an excessive expansion and dysfunction of adipose tissue. It also causes an inflammatory response and excessive accumulation of the extracellular matrix in adipose tissue, which is responsible for adipose tissue remodeling [29].

We can divide the genetic factors that underlie the substrate of obesity into syndromic and non-syndromic. A brief characterization of the genetic basis of obesity is described in Table 1.

**Table 1:** Genetic causes of obesity.

Genetically Related Types of Obesity	Cause of Obesity	The Syndrome or Gene Symbol	Ref.
**Syndromic obesity**	Associated with other genetic abnormalities and developmental defects of organs/systems	Prader–Willi syndrome, Albright syndrome, Down syndrome, Bardet–Biedl syndrome, Alström syndrome, Cohen syndrome, Fragile X syndrome, Monosomy 1p36 syndrome, Proximal 16p11.2 microdeletion syndrome	[30,31]
**Non-syndromic obesity**			
Monogenic obesity	Caused by single gene mutation	*LEP*, *LEPR*, *POMC*, *SIM1*, *PCSK1*, *MC4R*	[28,30,32,33]
Polygenic obesity	Associated with involvement of many genes whose function is modulated by environment	*FTO*, *MC4R*, *GNPDA2*, *BDNF*, *SH2B1*, *KCTD15*, *TMEM18*, *NEGR1*, *TLR4*, *TLR9*, *GPDIL*	[30,32]

### 3.1. Syndromic Obesity

Syndromic obesity is associated with a genetic syndrome in which obesity is one element of the clinical phenotype. It co-occurs with disorders such as intellectual disability, dysmorphic features, organ disorders, or specific behavioral disorders [30,34,35]. It is usually defined as early-onset obesity. Syndromic obesity can be caused by genetically determined dysmorphic syndromes, chromosomal aberration syndromes, microdeletion syndromes, and microreplication syndromes including genetic diseases determined by parental genomic stigma [30].

Approximately 140 genetic syndromes are currently known to be associated with syndromic obesity, one of the most common being Prader–Willi syndrome, which has a prevalence of 1 in 10,000 [36]. Inheritance in syndromic obesity can be both autosomal dominant and recessive and can be associated with chromosomal aberrations or coupled to the X chromosome or be associated with fragment copy number variations (CNVs). In addition, in some genetic syndromes, a similar clinical phenotype of a diverse genetic background is observed, so the diagnosis of this type of obesity requires the collaboration of multiple specialists especially in the field of genetic diagnoses [30]. Table 2 shows some of the genetic syndromes present with syndromic obesity.

**Table 2:** Some genetic syndromes with syndromic obesity.

Obesity-Related Syndrome	Gene/ Chromosome	Inheritance Pattern	Clinical Features	Obesity Features	Ref.
Prader–Willi Syndrome (PWS)	Chromosomal disorder with region on chromosome 15q11.2-q1	AD	Endocrinopathies, hyperphagia, mild dysmorphic features, intellectual disability, severe hypotonia, developmental delay, intellectual disability, small hands and feet, characteristic behavior (e.g., skin picking, outbursts, anxiety)	Hyperphagia onset around age 8 results in obesity in absence of it if not controlled	[30,34,36,37].
Alström Syndrome	Mutations in *ALMS1* gene (2p13)	AR	Insulin resistance, type 2 diabetes, hearing loss, cone-rod dystrophy, non-alcoholic fatty liver, chronic progressive kidney disease	Truncal obesity developed during first year of life	
Fragile X Syndrome (FRAX)	Triplet repeat expansion of CGG repeats greater than 200 in size in 5′ untranslated region of *FMR1* gene (Xq27.3)	X-linked	Autism spectrum disorder, intellectual disability, mild dysmorphic features, behavioral concerns, sleep disturbances, hypotonia, gastroesophageal reflux, scoliosis	Obesity and excessive appetite	
Down Syndrome	Trisomy 21, Robertsonian translocations, and mosaicism involving chromosome 21	AD	Intellectual disability, dysmorphic features, developmental delay, intellectual disability, characteristic facial features, hypotonia, heart defect, short stature, hypothyroidism, leukemia	Obesity	
Bardet–Biedl Syndrome	Mutations in genes *BBS1-BBS21/C80RF37*, *SCAPER*, *SCLT1*, *CEP164*	AR, oligogenic inheritance suggested in some families	Retinal cone-rod dystrophy, eye anomalies, polydactyly, hypogonadism, anosmia, renal malformations, behavioral concerns	Central obesity develops in first year of life	
Cohen Syndrome	Mutation of vacuolar protein sorting 13 homolog B (*VPS13B*) gene 8q22.2	AR	Failure to thrive in infancy and childhood; early-onset hypotonia; developmental delays; microcephaly; psychomotor retardation; neutropenia, progressive retinochoroidal dystrophy and myopia; joint hypermobility; characteristic facial features; a cheerful disposition	Obesity of trunk appearing in mid-childhood or later	
Smith–Magenis Syndrome	Deletion 17p11.2, RAI1	AD	Childhood-onset abdominal obesity, feeding difficulties, hypotonia, developmental delay, sleep disturbances, behavioral abnormalities, self-injurious behaviors, cognitive impairment	Childhood-onset truncal obesity	
Kallmann Syndrome	*ANOS1*, *KAL1*, *FGFR*, *FGF8*, *PROKR2*, *PROK2*	X-linked recessive pattern and autosomal recessive or dominant pattern with incomplete penetrance	Variable combination of hypogonadotropic hypogonadism and anosmia	Obesity reported in *PROKR2* and *KAL1*	

AD—Autosomal dominant, AR—Autosomal recessive.

Genetic syndromes associated with obesity whose genetic basis is well understood in addition to Prader–Willi syndrome (PWS) include Cohen syndrome, Bardet–Biedl syndrome, and Alström syndrome [35,37].

Weight gain in syndromic obesity is associated with disorders of energy homeostasis that increase BMI and are caused by hyperphagia, insulin resistance, or other endocrine disorders such as hypothyroidism [30]. The course of the disease varies in different genetic syndromes accompanied by obesity, and knowledge of these facts should be considered in prevention and dietary treatment, which should include the selection of the most beneficial nutrients.

In children with PWS, after a period of poor appetite in infancy between 4 and 8 years of age, increased appetite and excessive weight gain occur, which is related to an abnormal perception of the feeling of satiety. Mealtimes are prolonged and, in addition, the feeling of hunger occurs 30 min after finishing eating. The development of obesity in PWS patients is further favored by a reduced caloric intake of up to 60% relative to healthy individuals, reduced awareness of overeating due to a low perception of gastric pain, lack of discomfort after overeating, and lack of dietary control, and is due to eating-related behavioral disturbances and lack of physical activity. The cause of hyperphagia in these patients is complex and related to an abnormal regulation of neurotransmitters in the hypothalamic centers of hunger and satiety. The key gene responsible for hyperphagia in PSW is currently reported to be *SNORD-116*, encoding snoRNA-small nuclear RNA (snoRNA) [30]. The best therapeutic strategy for patients with PWS is a reduction diet, restricting access to food, and incorporating physical activity. A dedicated nutritional pyramid for PWS patients is based on 6–8 portions of vegetables per day, 4 portions of fruit, 3–5 portions of cereal products, 2 portions of protein, and 2 of dairy. Daily calorie intake should not exceed 1200 kcal/day [38].

In Alström syndrome, hyperphagia occurs as early as infancy and is accompanied by hyperinsulinemia and hyperglycemia, leading to the development of type 2 diabetes and moderate abdominal obesity.

Some patients with Fragile X chromosome syndrome (FRAX) develop excessive appetite with obsessive behaviors that lead to food seeking and consequently obesity [30,39,40]. Interestingly, obesity will also appear in individuals with deletions involving the *FMR1* gene.

In contrast, children with trisomy 21 show elevated leptin levels, reduced resting energy expenditure, and reduced physical activity due to muscular hypotonia and, in addition, hypothyroidism may contribute to obesity [30].

### 3.2. Monogenic Obesity

Control of energy balance depends on a properly functioning leptin–melanocortin pathway in the hypothalamus. Therefore, mutations in the leptin (*LEP*), leptin receptor (*LEPR*), proopiomelanocortin (*POMC*), single-minded homolog-1 (*SIM1*), prohormone convertase subtilisin/kexin type 1 (*PCSK1*) genes, melanocortin receptor 4 (*MC4R*) brain-derived neurotrophic factors (*BDNFs*), and neurotrophic tyrosine kinase receptor type 2 gene (*NTRK2*) are associated with the occurrence of monogenic obesity. This type of obesity is associated with the presence of mutations in single genes and results from their malfunction. [30,34,41]. Mutations in this type of obesity are characterized by severe early-onset obesity accompanied by endocrine disorders and abnormal eating behaviors. Inheritance in this type of obesity is usually autosomal recessive or dominant [41]. Monogenic forms of obesity affect approximately 5% of patients [42,43]. However, it is a clinical challenge to treat patients with monogenic non-syndromic obesity due to the complex phenotypes present. These patients present with morbid obesity that is refractory to classical treatments [41].

### 3.3. Polygenic Obesity

Polygenic obesity, also known as common obesity, can be caused by a variety of genetic variants both in mutations and polymorphic variants that occur in several genes. Such a genotype results in increased individual susceptibility to environmental factors affecting obesity. It is characterized by a later onset of obesity and reduced severity compared to monogenic obesity [44]. The development of both next-generation sequencing (NGS) and genome-wide association studies (GWASs) has provided insight into the molecular basis and identification of genetic associations in obesity. In total, more than 600 genes associated with obesity in humans have been selected. About 30 neuroendocrine peptides are also known to influence eating behavior, although only ghrelin appears to play an important role in appetite regulation and energy balance [34].

Among the genes associated with susceptibility to obesity, three groups of genes can be distinguished that affect energy balance. The first includes genes affecting the regulation of food intake, the second area is genes involved in adipogenesis including triglyceride storage, and the third area is genes related to energy expenditure and adaptive thermogenesis [27]. Table 3 gives examples of genes associated with obesity by classifying them into the appropriate group concerning phenotypic characteristics.

#### 3.3.1. FTO Gene

Variants in the obesity-associated gene *FTO* are suspected of being associated with obesity risk [41]. *FTO* variant homozygotes have been shown to have an average of 3 kg more body weight, in contrast to individuals without these alleles [30].

Polymorphic variants located in intron 1 of the *FTO* gene have been confirmed to affect its expression. In contrast, the *FTO* protein influences adipogenesis by increasing the abundance of the adipogenic regulatory factor RUNX1 1 (RUNX1T1). *FTO* controls RUNX1T1 splicing by regulating m6a and thus *FTO* directly modulates obesity at the m6A level. SNPs located in intron1 of *FTO* have also been shown to affect the expression of neighboring genes such as Iroquois homeobox 3 (IRX3), Iroquois homeobox 5 (IRX5), and RPGR-Interacting Protein 1-Like (RPGRIP1L); another possible role of *FTO* in obesity is the regulation of macronutrient intake, as the *FTO* gene is expressed in the hypothalamus during starvation. This demonstrates the role of *FTO* in regulating food intake. The impact and role of specific SNPs are not entirely clear, although a general effect of *FTO* gene polymorphisms on BMI and body composition has been established [44,48].

The A allele of the rs9939609 variant of the gene *FTO* is most commonly associated with weight gain and obesity, and also influences a higher risk of type 2 diabetes [49]. A study on a homogeneous Polish population showed that the effect of SNPs in the first intron of the *FTO* gene on obesity is modulated by age and sex; this was particularly noticeable in men aged 45–50 years [50]. In some populations, the effect of *FTO* polymorphisms on BMI may also be masked by dietary habits and physical activity, which may modulate the effect of *FTO* polymorphisms on obesity, due to the fact that increased physical activity reduced the effect of the A allele of rs9939609 on BMI [44].

#### 3.3.2. PLIN1 Gene

Perilipin 1 (*PLIN1*) is the best-characterized member of the perilipin family of proteins. In energy metabolism, they are responsible for controlling access to triglyceride stores in adipocytes [45,51]. In the fed state, Perilipin 1 restricts lipase access to stored triglycerides, while in the fasting state, it stimulates hormonal lipolysis. The abnormal regulation of lipolysis, in which there is an excessive release of fatty acids from adipose tissue, often accompanies obesity, which may also contribute to insulin resistance and type 2 diabetes [45].

An analysis of several SNP variants (rs2289487, rs1561726, rs2304794, rs894160, rs2304795, rs1052700) in the *PLIN1* gene was conducted to assess their significance in obesity on weight loss and glucose metabolism [45,51]. Genotyping was conducted on large multi-ethnic populations, which showed gender-specific associations between two SNPs (rs2289487 and rs894160) and anthropometric and metabolic traits including plasma glucose and triglyceride concentrations. A lower risk of obesity for variants of both polymorphisms has been shown in women. The *PLIN1* Allele C gene rs2289487 variant has also been shown to enhance weight loss, and has been shown to reduce insulin resistance and glucose levels after dieting with a hypocaloric diet [52]. A single study has shown that an estrogen receptor-related receptor activates the transcription of the *PLIN1* gene [45]. Therefore, the relationships between gender, obesity, and the metabolic consequences of obesity should be considered [53].

Another study of the rs1052700 variant of the PLIN1 gene showed an association with increased obesity in the Caucasian population [54].

#### 3.3.3. SIRT1-7 Gene Family

Sirtuins (*SIRT1-7*) belong to a family of conserved NAD +-dependent protein deacetylases. The role of these genes in adipose tissue remodeling is associated with adipocyte, lipid mobilization inflammatory changes, and adipose tissue fibrosis [29]. Pathological adipose tissue expansion is associated with mitochondrial dysfunction, which affects lipid metabolism, adipocyte differentiation, insulin sensitivity, and thermogenesis. Mitochondria appear to be potential targets for obesity therapy in metabolic diseases. The regulation of mitochondrial biogenesis, mitochondrial autophagy, and mitochondrial translocation may be targets for the development of pharmacotherapy based on mitochondrial pathways [29,55].

Sirtuin 3 (*SIRT3*) is a mitochondrially localized deacetylase belonging to the sirtuin family. The role of *SIRT3* is related to the activation of mitochondrial function and contributes to adaptive thermogenesis [46].

The *SIRT3* gene is located in chromosome 11p15.5 in a region that is associated with longevity. In obesity studies, it has been shown that *SIRT3* can act as a positive regulator of insulin sensitivity, while *SIRT3* expression is down-regulated in obesity. In pathological conditions, *SIRT3* levels are reduced, as is the case with a high-fat diet. It has been reported that dietary calorie restriction attenuates the age-dependent decline in *SIRT3* levels [56].

### 3.4. Vitamin D Receptor Gene

Different studies have shown that adipose tissue function depends on vitamin D [57]. The vitamin D receptor (VDR), found in many tissues including adipose and bone tissue, controls serum vitamin D levels. The activated form of vitamin D in combination with the VDR receptor can affect the initiation and transcription of many genes. It is estimated that up to 500 genes may be affected [58]. Genetic variants of the *VDR* are also significant for weight gain and the development of obesity, and may also affect both the activity of the receptor itself and serum vitamin D levels [57,58].

The *VDR* gene has numerous genetic variants; 506 variants have been designated as clinically relevant variants [59]. Polymorphic variants of the *VDR* gene are responsible for, among other things, the stability of nascent mRNA: rs7975232 (ApaI), rs731236 (TaqI), and rs1544410 (BsmI). On the other hand, variants rs2228570 (FokI), rs731236, and rs11568820 have been assessed to be related to vitamin D levels [60,61]. Data based on meta-analyses indicate that rs731236 and rs2228570 variants are associated with a better response to vitamin D supplementation and may modulate the response to supplementation [62]. In addition, it has been shown that the T allele of rs2228570 may be a risk factor for obesity while the T allele of rs731236 may have a protective effect [63]. Genetic variation within the *VDR* gene is a significant factor, associated with anthropometric characteristics in obesity in a central European population [64]. Increased susceptibility to obesity has also been shown by some researchers for the rs1544410 and rs731236 variants in the *VDR* gene [52,65,66]. The heterozygous rs731236 variant of the *VDR* gene has also been shown to have a protective effect on the osteoporosis phenotype accompanied by increased BMI among women with osteoporosis [67].

However, some reports do not confirm these data and perhaps it is related to the size of the study group, ethnicity, or metabolic health including comorbidities [63,67].

### 3.5. MCM6 Gene Associated with Lactose Intolerance

The rs4988235 polymorphic variant is often used as a predictor of dairy consumption. It is associated with lactose tolerance (LP) mainly in individuals of the European population, although not exclusively [68]. The rs4988235 variant is positioned 14 kb upstream of the *LCT* gene and is located in intron 13 of the *MCM6* gene (component 6 of the minichromosome maintenance complex). Its function is related to the transcriptional activation of the *LCT* lactase gene promoter. A homozygous arrangement of A alleles enables lactose digestion (LP), while a homozygous arrangement for G alleles is associated with lactose intolerance (LNT). Heterozygotes carrying the AG allele have an intermediate phenotype, although they are considered to digest lactose (LP) [69].

The rs4988235 polymorphic variant showed a significant association with BMI and fat mass, confirming its association with obesity risk [70,71]. A meta-analysis of nearly 2 million participants showed an association of the A allele for the rs4988235 variant with higher milk consumption and higher BMI. At the same time, they showed lower levels of both total cholesterol (TC) and serum LDL and HDL concentrations. These patients also had a lower risk of developing ischemic heart disease. Thus, it seems that the A allele for the rs498823 variant does not affect the risk of cardiovascular disease and does not require limiting milk intake, but may affect weight gain [71].

A study in a population of postmenopausal women found that carrying the G allele for the rs4988235 variant of the *MCM6* gene significantly increased the risk of developing T2DM and loss of femoral neck BMD mineral density with age [69]. Interestingly, in people with lactose intolerance (LNP), higher milk consumption was shown to be associated with a lower risk of type 2 diabetes, which may be related to changes in the gut microbiota. Increased bacterial strains of *Bifidobacterium* and decreased amounts of *Prevotella* are associated with circulating metabolites including increased indolopropionate and decreased amino acid metabolites [72]. The beneficial effect of milk intake also depended on metabolites derived from GAA (-glutamyloline) and tryptophan (indolopropionate) BCAAs (-hydroxycaptopropionate and a-hydroxyisovate) [73]. This would confirm the relationship between milk intake and the involvement of the gut microbiota and circulating metabolites, ultimately resulting in adaptation to lactose intake in patients with the genotype responsible for lactose intolerance [69,72]. Milk consumption in these individuals may reduce the risk of type 2 diabetes [72].

### 3.6. Diagnosis of the Genetic Basis of Obesity

#### 3.6.1. Polygenic Obesity

The development of genetic diagnostic methods has opened up many new opportunities to learn about, personalize, or develop individualized dietary management based on genetic profiling. The evaluation of both genetic predisposition and the cause of genetically determined obesity makes it possible to find out the genetic basis of obesity in the patient under study. This is mostly based on NSG genomic panels [74,75]. However, there is a lack of guidelines that can clearly define a regimen for management of patients in whom variant lesions of uncertain pathogenicity (VUS) are found or the interpretation for variants is not clear [75].

#### 3.6.2. Monogenic Obesity

Monogenic obesity is suspected in children with an early onset of weight gain (<2 years of age) and concurrent hyperphagia. It is estimated that it will be diagnosed in 3–10% of children with grade III obesity. Additional symptoms that may indicate monogenic obesity include somatomegaly, an increased head circumference, hyperinsulinemia, hypogonadotropic hypogonadism, GH deficiency, immune deficiencies, hypothyroidism, autism, behavioral problems, insulin resistance, a slow heart rate, a low basal metabolic rate, intellectual disability, hyperactivity, severe insulin resistance, diarrhea in the neonatal period, hypoglycemia, hypothyroidism, adrenal insufficiency, and central uremia [76].

The diagnosis should be confirmed by genetic testing based on genome sequence evaluation. Genomic panels based on next-generation NGS sequencing evaluating the following genes are available: *ADCY3*; *ADRB2*; *ADRB3*; *AFF4*; *AGRP*; *ALMS1*; *ARL6*; *BBS1*; *BBS10*; *BBS12*; *BBS2*; *BBS4*; *BBS5*; *BBS7*; *BBS9*; *BDNF*; *CARTPT*; *CCDC28B*; *CELA2A*; *CEP19*; *CEP290*; *CPE*; *CUL4B*; *DYRK1B*; *ENPP1*; *FTO*; *GHR*; *GHRL*; *GNAS*; *GNB3*; *HDAC4*; *HDAC8*; *INPP5E*; *KIDINS220*; *KSR2*; *LAS1L*; *LEP*; *LEPR*; *LZTFL1*; *MAGEL2*; *MC3R*; *MC4R*; *MKKS*; *MKS1*; *MRAP2*; *NR0B2*; *NTRK2*; *PCSK1*; *PHF6*; *PHIP*; *POMC*; *PPARG*; *PYY*; *SDC3*; *SDCCAG8*; *SH2B1*; *SIM1*; *SLC6A14*; *TRIM32*; *TTC8*; *TUB*; *UCP2*; *UCP3*; *VPS13B*; and *WDPCP* [75,77].

The evaluation of deletions and duplications, in regions associated with obesity, is possible by assessing genes *LEPR*, *POMC*, *SIM1*, *LEP*, *MC4R*, *MC2R*, and *MC3R*, and in the 16p11.2 region by MLPA [78].

Since personalized treatment is available for some mutations, an important aspect is to determine the genetic background to personalize treatment [76].

#### 3.6.3. Syndromic Obesity

It is estimated that syndromic obesity can be a characteristic feature of almost 100 genetic syndromes and its cause can be either a mutation in a single gene or a change involving multiple genes in a chromosomal region. The genetic diagnosis should be personalized for the selected genetic syndrome based on methods of classical cytogenetics, aCGH, FISH, MLPA, or molecular NGS [76].

## 4. Genetic Determinants of Dietary Choices

### 4.1. Genetic Influences on Taste

Taste is one of the five traditional senses. Perceived tastes have been classically categorized into five basic ones: salty, sweet, bitter, sour, and umami or savory. However, taste is not the only factor regulating food intake. It is one of the most important factors that influence food choices. Taste is perceived individually; it is influenced by genetic differences in taste receptors, and the distribution of taste papillae, which do not perceive only taste, but also temperature, touch, or nociception. The composition of saliva and the sensitivity of taste receptors are also important [79].

Preference for sweet taste and high-fat foods decreases with an increased perception of bitter taste [80]. A higher perception of bitter taste is associated with BMI, obesity, and cardiovascular risk factors. The perception of bitter taste and a preference for high-fat foods guide food choices, which are linked to obesity [81]. These traits may be partly genetically determined [80].

The sense of bitter, sweet, and umami tastes are mediated by G-protein-coupled receptors (GPCRs). Bitter taste receptors are encoded by *the TAS2R* gene family including 25–30 genes located on chromosomes 12p13, 7q34, and 5p15.31 [80]. Bitter taste perception is influenced by two common haplotypes, one being *TAS2R38*, which affects bitter taste sensitivity and perception through the bitter compounds phenylthiocarbamide, propylthiouracil *(PROP*), and thiocyanates, contained in vegetables such as Brussels sprouts and broccoli [82]. People most sensitive to *PROP* are less likely to like bitter fruits and vegetables such as grapefruit and kale. Therefore, in this group of people, low-energy foods can be replaced by high-energy foods [83]. It has been suggested that the *TAS2R38* haplotype may be a predictor of obesity; however, larger cohort studies have not confirmed an association between *TAS2R38* and obesity despite the association of this polymorphism with nutrition [80,82,84]. Individuals with *TAS2R* variants *TAS2R38*, *TAS2R5*, and *TAS2R16* may be characterized by behaviors such as an increased intake of sugary and high-fat foods or reduced vegetable intake. These characteristics may be associated with the choice of highly nutritional foods, a high consumption of which can lead to obesity [80].

### 4.2. Genetics of Weight Gain

Variants in the obesity-associated gene *FTO* are suspected of being associated with obesity risk [85]. Homozygotes of the *FTO* variant have been shown to have an average of 3 kg more body weight, in contrast to individuals without these alleles [49]. The association between diet and *FTO* has been widely studied. Increased energy, fat, and protein intake have been associated with variation in *FTO*. The consumption of energy-rich or high-fat products presented by the holders of this variant of the *FTO* gene can favor excessive energy supply, which in turn can lead to obesity [85]. However, it has been shown that increased physical activity and a healthy diet can reduce the effect of *FTO* loci on obesity risk by 30–40% [42].

Another gene suspected of influencing the incidence of obesity and higher BMI is the melanocortin-4 receptor gene (*MC4R*). The C allele variant rs17782313 gene *MC4R* is thought to be a risk factor for obesity. It was shown that homozygotes of TT variant rs17782313 gene *MC4R* have significantly lower appetite compared to allele CT or CC carriers [86].

### 4.3. Impact of Diet on Weight Gain

The science that focuses on the study of the influence of genetic variation on dietary response is nutrigenomics. The research carried out in this field makes it possible to tailor a patient’s diet according to their genetic needs. In recent years, nutrigenomics research has made it possible to identify genetic variants associated with susceptibility to various diseases, among them obesity, by interacting with a dietary factor [87].

For the allele A rs9939609 *FTO* gene, individuals who consume high amounts of fat in their diet will have a higher risk of obesity. Similarly, for the allele A variant rs8050136 *FTO* gene, a high-carbohydrate diet will be associated with an increased risk of obesity [87]. In contrast, it has been shown that for some *FTO* genotypes, a high-protein diet can reduce the risk of obesity and increase weight loss [85,87]. Also, for the allele T rs4988235 *LCT* gene, the consumption of large amounts of dairy products, and for the allele G rs1801282 *PPARG* gene, a high-fat diet, will increase the risk of obesity [87]. The effect of dietary intervention on allelic variants is shown in Table 4.

## 5. Epigenetics

Dietary patterns have a significant impact on gene expression. Individuals whose diets were characterized by processed meats, desserts, sweets, and high amounts of refined cereal products showed the expression of genes responsible for the inflammatory response compared to those whose diets included high amounts of vegetables, fruits, and whole-grain cereal products [102]. Diets high in fat, especially in saturated fatty acids, induct gene expression profiles that are related to glucose intolerance, liver lipid accumulation, inflammation, and an increased expression of neuropeptides involved in obesity development [87]. It was also shown that apple polyphenols may prevent obesity induced by diet by regulating genes involved in lipolysis, adipogenesis, and fatty acid oxidation [103,104].

Dietary bioactive components are also mentioned. They may have an obesity-preventing effect. These include resveratrol, a non-flavonoid polyphenol produced by several plants in response to injury or a fungal attack [104,105]. It was shown that obese subjects taking 150 mg/day of resveratrol for one month significantly decreased their metabolic rate of sleep with no change in 24 h energy expenditure. The 24 h respiratory quotient also increased, especially during the day and after feeding. This suggests an improvement in metabolic efficiency. That effect was compared to a calorie-restricted diet or resistance training [106].

It was suggested that dietetic interventions including supplying phytochemicals found in cruciferous vegetables or green tea may prevent or reverse epigenetic changes in age-related disease and may play an anticancer role [107]. It was also suggested that bioactive compounds like epigallocatechin gallate (EGCG), the strong anti-inflammatory agent found in large amounts in green tea, may be preventive in cancer and disorders inducing histone modifications and obesity [108]. Products of soybeans rich in proteins that contain isoflavones, genistein among them, have been shown to alter the risk of obesity in non-human primate models by epigenetic modifications [109]. An anti-obesity effect was suggested with dietary information on sulforaphane by suppressing lipogenesis and inhibiting adipogenesis [110].

The link between obesity and vitamin D deficiency was also detected. 1,25(OH)_2_D_3_ is a fat-soluble vitamin whose deficiency is associated with obesity, among others. It is important for Ca^2+^-Pi and glucose metabolism in the adipocytes among obese individuals. Furthermore, 1,25(OH)_2_D_3_ regulates the expression of genes associated with adipogenesis in mature adipocytes [111]. A vitamin D receptor (VDR) was reported to occur in most human cells. It was shown that VDR is abundantly expressed in adipose tissue, and it plays a crucial role in energy metabolism and adipogenesis. Moreover, the VDR expression decreases with the adipocytes’ differentiation progress, which influences the obesity-related risk [112,113]. Vitamin D may exhibit anti-adipogenic effects by inhibiting the differentiation of human adipose-derived mesenchymal stem cells (hADMSCs) in adipose tissue cells. This is accomplished by the suppression of factors such as CCAAT-enhancer-binding proteins (C/EBPα and C/EBPβ) and peroxisome proliferator-activated receptor-gamma (PPARγ), which are key in adipocyte formation. The effects of Bisphenol A (BPA) on obesity have also been reported. BPA is an endocrine disruptor that is involved in everyday life, among other things, by being found in products containing polycarbonate plastics. Bisphenol A has been shown to promote adipogenesis by affecting the expression of genes responsible for the differentiation of adipose tissue cells, while at the same time increasing adipocyte formation by increasing the accumulation of lipid droplets in hADMSCs. Vitamin D acts antagonistically to BPA, suggesting that exposure to BPA may reduce the anti-adipogenic effect of vitamin D [114].

The association of vitamin B12 and folate on obesity has also been demonstrated. It has been suggested that an adequate supply of these vitamins may promote the methylation of genes that are important in the development of obesity including *FTO* and *LEP.* Proper methylation has the potential to reduce the risk of obesity by supporting proper gene function. It was shown that children whose diets contained enough of these nutrients showed more favorable methylation patterns in genes related to obesity, suggesting that these substances may have a protective effect against obesity [115].

### Fetal Programing

The term “fetal programming” was introduced in the early 1990s by Dr. David Barker as the concept of fetal origins of adult disease (FOAD) [116]. The prenatal influence on humans appears to be dependent on maternal body composition, stress, metabolism, and diet from conception. Also, paternal influences are being recognized. Thus, the lifestyle of parents appears to have an impact on the health of offspring before birth via fetal programming. Mechanisms of epigenetic modification enabled an increased understanding of fetal programming and how environmental, epigenetic, and genetic factors relate to cause lasting effects on offspring adiposity and future metabolic outcomes [117].

There is also a link between microbiome disruption in maternal obesity, use of antibiotics while pregnant, a cesarean section, and early infancy and increased childhood obesity risk. Changes in microbiome colonization in the early period of development can increase the occurrence of asthma, diabetes, allergy, and obesity. The correct nutrition of the mother prior to and during pregnancy is crucial for the offspring’s long-term health. Fetal undernutrition was shown to be associated with a higher risk of diseases, among them central obesity and diabetes [117]. Malnutrition in pregnancy also plays a crucial role in fetal programming. During the famine in the Netherlands at the end of World War 2, there was a significant reduction in calorie intake. Children of women who were pregnant during the approximately six-month famine were studied. It was observed that, due to inadequate caloric coverage, the following were affected by malnutrition, depending on the time of pregnancy during which malnutrition occurred: the atherogenic lipid profile, coronary heart disease, and obesity—early in pregnancy; microglobulinemia and obstructive respiratory disease—mid-pregnancy; and, irrespective of the time of pregnancy, impaired glucose tolerance [118]. Research in epigenetics and fetal programming allows us to understand the mechanisms involved in diseases of civilization, among them diabetes and obesity. Unfortunately, many of the mechanisms involved in fetal programming are still unknown [119].

In summary, several key periods in human development have been identified during human development in which epigenetic changes occur, and the prenatal period during which intensive fetal growth occurs. It has been shown that factors that influence the function of metabolic pathways in the body include elements such as maternal nutrition; both malnutrition and malnutrition overeating can lead to obesity and the development of type 2 diabetes. Maternal exposure to toxins including smoking also causes epigenetic modulations. Maternal diabetes and a so-called ‘unhealthy diet’ rich in sugary drinks, and fried foods rich in unsaturated fats, may also be a reason for the epigenetic changes leading to obesity [49,50,51,52].

## 6. Perspectives, Challenges, and Future Directions in Obesity Research

**Figure 3:**
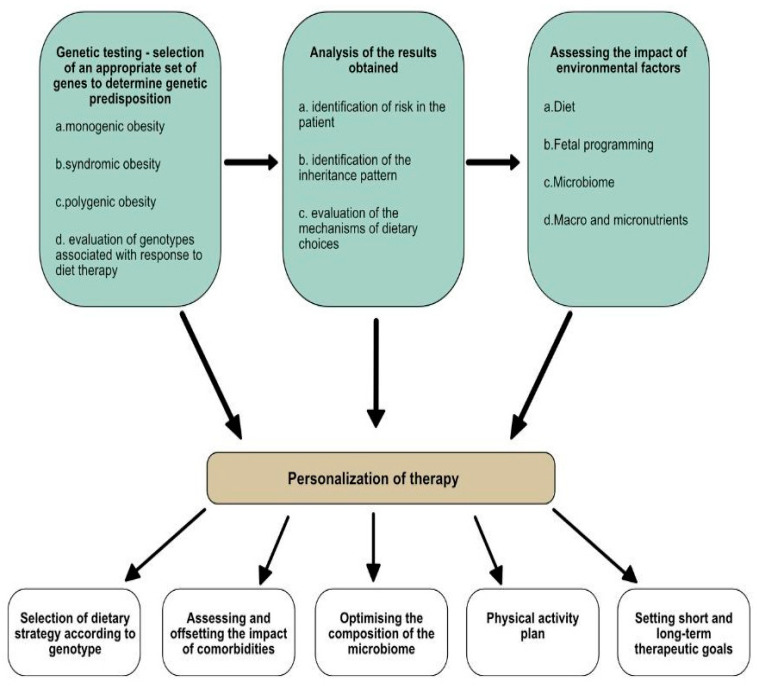
The personalization of the therapeutic management of patients with obesity. In the process of personalizing obesity treatment therapy, according to the scheme presented, a holistic approach to the patient is necessary. Achieving positive and lasting treatment results requires the cooperation of various medical specialists, such as a doctor, geneticist, nutritionist, or physiotherapist. The personalization of therapy includes genotype-specific diet selection, reduction in comorbidities, the optimization of the microbiome, establishment of a physical activity plan, and setting therapeutic goals.

This review indicates that the onset of obesity is influenced by individual factors, particularly genes, which play a significant role. Additionally, genes affect personal dietary preferences, which in turn influence gene expression. Yet, obesity is also governed by environmental factors such as a high-calorie diet and/or lack of physical activity. Thus, an understanding of obesity as a multifactorial disease encourages comprehensive strategies in diagnostics and management (Figure 3).

The expression of obesity-related genes can be influenced by various factors, including age, sex, hormonal changes, and epigenetic modifications. This variability complicates the development of targeted treatments or interventions based solely on genetic information, as the same genetic variants might have different effects in different populations or under different conditions. A limited number of genes are closely linked to a high risk of obesity, referred to as monogenic obesity [32,120]. The incorporation of technology in weight management efforts is vital in cases of chromosomal disorders or insertion–deletion mutations, so we consider these diagnostic criteria in the management of obesity. Collaborative efforts with diagnostic companies that produce genetic testing kits could further pave the way for personalized approaches in obesity therapy [74,77,78]. The research, which focuses on restoring energy balance by reducing food intake and increasing energy expenditure, provides evidence efficacy and safety [121]. This could represent a promising targeted molecular treatment for monogenic obesity. Specifically, gene therapy aimed at the hypothalamic *BDNF* gene, utilizing an autoregulatory AAV vector, effectively tackles obesity and metabolic disorders associated with *MC4R* deficiency in a clinically relevant mouse model [122]. Furthermore, continued investigation into the mechanisms underlying these genetic interventions may lead to innovative treatment protocols. Moreover, an emphasis should be placed on early intervention strategies that affect maternal and child health [123]. Initiatives designed to improve prenatal nutrition and promote healthy lifestyles during critical growth periods may significantly reduce obesity risk in future generations [124]. This perspective highlights the need to consider long-term health implications from conception onward, promoting preventative measures that target maternal health and nutrition [125]. However, behavioral change is often challenging to sustain.

In cases of mismatch disorders, individual variability in gene expression influenced by environmental factors complicates the understanding of the etiology of obesity [126]. Such variability highlights the need for tailored interventions that recognize the unique genetic and environmental contexts of individuals [127]. To accurately assess the risk and development of obesity, studies must take population-specific variability into account. Critical research is needed to focus on diverse cohorts to better understand the complex interplay of genetics, environment, and lifestyle in obesity. For instance, the *FTO, PPARG, MC4R,* and *LEPR* genes have shown significant correlation with obesity in European populations [128,129], while the association appears to be weaker in some Asian populations, including oceanic groups (for *FTO* and *PPARG*), as well as in African populations (for *MC4R* and *LEPR* genes) [130,131,132,133,134]. Variability in these associations suggests that genetic factors may interact with different environmental influences, lifestyle choices, and cultural dietary practices. Sociocultural influences, convenience of unhealthy food options, and lifestyle habits contribute to the difficulty in maintaining weight loss and adopting healthier eating patterns over time. Whether due to cultural norms, emotional eating, or the influence of highly palatable foods, it is vital to find effective strategies for promoting long-term adherence to healthy behaviors in diverse populations. Understanding these relationships allows for the development of personalized nutrition strategies that can effectively mitigate obesity risk. Future research should focus on developing personalized nutrition plans based on an individual’s genetic make-up, lifestyle, and environmental influences. Advanced technologies like genomics, metabolomics, and machine learning could aid in creating personalized dietary recommendations that optimize weight management. What is more, mobile applications, wearable devices, and tele-health platforms can facilitate continuous monitoring and provide support for individuals pursuing healthier lifestyles including diet. Additionally, research should investigate the impact of novel dietary components, such as bioactive compounds, on gene expression and obesity prevention. What is more, access to healthy foods and opportunities for physical activity, particularly in lower-socioeconomic populations, amplify the urgency of addressing these disparities. People in resource-limited settings may struggle to implement suggested dietary changes, and strategies that do not address these challenges may have limited effectiveness. Barriers to healthful living, such as food deserts and limited recreational spaces, exacerbate the risk of obesity and further complicate efforts to combat this epidemic. Therefore, it is essential to advocate for policies that remove these barriers and promote equitable access to resources that enable healthy lifestyle choices for all communities, irrespective of their socioeconomic status.

Personalized nutrition, which takes into account a person’s genetic profile, metabolism, and lifestyle, is still emerging and is not yet widely available. Additionally, focusing solely on genetic and nutritional factors may ignore the psychological aspects of obesity [135]. Emotional eating, stress, and mental health issues heavily influence eating habits and physical activity [136]. Effective interventions must therefore incorporate behavioral elements, which can be difficult to implement and sustain. What is more, there is a need for a more integrative approach that combines genetic research with nutritional science and public policy. Current policy frameworks may fail to incorporate findings from obesity genetics, which could enhance targeted interventions and inform public health strategies. However, many current policies focus primarily on addressing calorie intake or physical activity levels without adequately considering the broader context of food systems, including agricultural practices, food marketing, and socioeconomic disparities [137].

## 7. Conclusions

In conclusion, addressing the complex issue of obesity requires a multifaceted approach that integrates genetic, nutritional, and environmental perspectives. There is a growing need for personalized interventions, longitudinal data collection, and community support mechanisms aimed at promoting sustainable lifestyle changes and improving health outcomes. Through collaborative efforts, researchers, healthcare providers, and policymakers can develop effective strategies to combat obesity and enhance public health.

## Figures and Tables

**Table 3:** Genes associated with obesity risk according to molecular background.

Function	Gene Name	Phenotypic Traits	Ref.
Genes encoding proteins that regulate food intake	*POMC*, *MC4R*, *LEP*, *LEPR*, *GHRL*, *PYY*, *ADCY3*	Relative hyperphagia Weight loss with energy restriction Good response to appetite-suppressant pharmacotherapy	[27,29,45,46,47]
Genes involved in adipogenesis, preadipocyte differentiation, triglyceride synthesis, regulation of lipid storage and lipolysis	*PPAR γ*; *[DGAT]-1*; *ADRB2*; *PLIN1*; *FTO*	No significant hyperphagia, worse response to nutritional intervention for selected SNPs	
Genes regulating mitochondrial biogenesis, influencing adaptive thermogenesis	*SIRT1-7*	Influence propensity to gain weight, modulate weight loss	

Genes: alpha-melanocyte-stimulating hormone [*POMC*], melanocortin-4 receptor [*MC4R*], leptin [*LEP*], ghrelin [*GHRL*], peptide YY [*PYY*], peroxisome proliferator-activated receptor-gamma [*PPAR γ*], diacylglycerol acyltransferase [[*DGAT*]-*1*], beta-adrenergic receptors [*ADRB2*], perilipin [*PLIN1*].

**Table 4:** Effect of dietary intervention depending on gene variant.

Type of Diet	Variant, Gene, Allele	Phenotypic Effect	Ref.
High protein	rs1558902, *FTO*, allele A	Greater weight loss	[88]
	rs987237, *TFAP2B*, allele G	Higher weight regains	[89]
	rs10830963, *MTNR1B*, allele G	Smaller weight loss in women	[90]
	rs12785878, *DHCR7*, allele T	Higher decreases in insulin and HOMA-IR	[91]
High fat	rs7903146, *TCF7L2*, allele T	Smaller weight loss and HOMA-IR	[92]
	rs3764261, *CETP*, allele C	Larger increases in HDL-c and decreases in triglycerides	[93]
	rs1440581, *PPM1K*, allele C	Smaller weight loss and smaller decreases in insulin and HOMA-IR	[94]
High carbohydrate	rs2943641, *IRS1*, allele C	Higher decreases in insulin, HOMA-IR, and weight loss	[95]
	rs236918, *PCSK7,* allele G	Higher decreases in insulin and HOMA-IR	[96]
Low fat	rs1558902, *FTO*, allele A	Less reduction in insulin and HOMA-IR	[97]
	rs964184, *APOA5*, allele G	Larger reduction in TC and LDL-c	[98]
	rs2287019, *GIPR*, allele T	Greater weight loss and greater decrease in glucose, insulin, and HOMA-IR	[99]
	rs2070895, *LIPC*, allele A	Higher decreases in TC and LDL-c and lower increase in HDL-c	[100]
Mediterranean diet	rs2069827, *IL6*, allele C	Lower weight gain	[101]

*APOA5*—apolipoprotein A5; *CETP*—cholesteryl ester transfer protein; *DHCR7*—7-dehydrocholesterol reductase; *FTO*—fat mass and obesity associated; *GIPR*—gastric inhibitory polypeptide receptor; HDL-c—high-density lipoprotein cholesterol; HOMA-IR—homeostasis model assessment of insulin resistance; *IL6*—interleukin-6; *IRS1*—insulin receptor substrate 1; LDL-c—low-density lipoprotein cholesterol; *LIPC*—lipase c, hepatic type; *MTNR1B*—melatonin receptor 1B; *PCSK7*—proprotein convertase subtilisin/kexin type 7; *PPM1K*—protein phosphatase, Mg2+/Mn2+-dependent 1K; TC—total cholesterol; *TCF7L2*—transcription factor 7-like 2; *TFAP2B*—transcription factor AP-2 beta.
